# Antischistosomal Properties of Hederacolchiside A1 Isolated from *Pulsatilla chinensis*

**DOI:** 10.3390/molecules23061431

**Published:** 2018-06-13

**Authors:** Naixin Kang, Wenhua Shen, Hongwei Gao, Yulin Feng, Weifeng Zhu, Shilin Yang, Yanli Liu, Qiongming Xu, Di Yu

**Affiliations:** 1 Department of Pharmacognosy, College of Pharmaceutical Science, Soochow University, Suzhou 215123, China; carnation518@hotmail.com (N.K.); yangshilin@suda.edu.cn (S.Y.); 2 College of Pharmaceutical Science, Jiangxi University of Traditional Chinese Medicine, Nanchang 330006, China; shwh94@outlook.com (W.S.); fengyulin2003@126.com (Y.F.); zwf0322@126.com (W.Z.); 3 College of Pharmacy, Guangxi University of Chinese Medicine, Nanning 530001, China; gaohongwei06@126.com; 4 Department of Pharmacology, College of Pharmaceutical Science, Soochow University, Suzhou 215123, China; 5 Department of Immunology, Genetics and Pathology, Science for Life Laboratory, Uppsala University, Uppsala 75105, Sweden; di.yu@igp.uu.se

**Keywords:** hederacolchiside A1, *Schistosoma japonicum*, *Schistosoma mansoni*, antischistosomal

## Abstract

*Background*: Schistosomiasis is a major neglected disease for which the current control strategy involves mass treatment with praziquantel, the only available drug. Hence, there is an urgent need to develop new antischistosomal compounds. *Methods*: The antischistosomal activity of hederacolchiside A1 (HSA) were determined by total or female worm burden reductions in mice harboring *Schistosoma japonicum* or *S. mansoni*. Pathology parameters were detected on HSA against 1-day-old *S. japonicum*-harboring mice. Moreover, we confirmed the antischistosomal effect of HSA on newly transformed schistosomula (NTS) of *S. japonicum* in vitro. *Results*: HSA, a natural product isolated from *Pulsatilla chinensis* (Bunge) Regel, was initially corroborated to possess promising antischistosomal properties. We demonstrated that HSA had high activity against *S. japonicum* and *S. mansoni* less in 11 days old parasites harbored in mice. The antischistosomal effect was even more than the currently used drugs, praziquantel, and artesunate. Furthermore, HSA could ameliorate the pathology parameters in mice harboring 1-day-old juvenile *S. japonicum*. We also confirmed that HSA-mediated antischistosomal activity is partly due to the morphological changes in the tegument system when NTS are exposed to HSA. *Conclusions*: HSA may have great potential to be an antischistosomal agent for further research.

## 1. Introduction

Schistosomiasis is a chronic and debilitating disease caused by digenetic trematodes from the genus *Schistosoma* mainly comprising three species: *Schistosoma japonicum*, *S. mansoni* and *S. haematobium* [1,2]. It is the second most frequent parasitic disease affecting humans after malaria, the global burden of schistosomiasis has been estimated to exceed 70 million disability-adjusted life years [3], but even the higher estimate might be an underestimation of the true burden [2,4,5]. According to the investigation of World Health Organization in 2012, around 800 million individuals are at risk of contracting the disease and 239 million people have been infected by schistosomes, whereas schistosomiasis has not evoked enough focus in this field [6,7]. Generally, human infections occur after penetration of the skin by the infectious larvae, or cercariae, and migration of the adult parasite to the portal vasculature surrounding the intestinal tract (*S. mansoni* and *S. japonicum*) or the vesicle plexus of the bladder (*S. haematobium*) [7]. Morbidity due to schistosomiasis includes hepatic and intestinal fibrosis (*S. mansoni* and *S. japonicum*), and ureteric and bladder fibrosis and calcification of the genitourinary tract (*S. haematobium*) [8].

As it stands now, the only clinical available drug against schistosomiasis is praziquantel, that has been used for 40 years [9]. Recently, a deluge of evidences indicated that the drug resistance of praziquantel has emerged in the clinic [10,11]. Praziquantel has little or no effect on eggs and immature worms [12], so pre-patent or newly acquired infections cannot be cured by praziquantel [9,13]. In order to provide new hit and lead compounds, the search for anthelmintic compounds from natural sources has intensified [14]. Natural products have been the source of medicines for thousands of years, and also provide modern medicine with effective pharmaceuticals for the treatment of diseases caused by parasites [15,16,17]. Artemisinin, isolated from the plant *Artemisia annua*, has been effectively used for schistosomiasis control [18], however, this drug is confined to the young developmental stages of the parasites.

*Pulsatilla chinensis* (Bunge) Regel, a traditional Chinese medicine with a long history, exhibits “blood-cooling” and detoxification activities. It has been widely used for adjunctive treatment of intestinal amebiasis, malaria, vaginal trichomoniasis, bacterial infections and malignant tumors [19,20,21,22]. Recent studies have found that various *P. chinensis* extracts and fractions had antiprotozoal activity against *Giardia intestinalis* [23] and selectively inhibited the growth of human intestinal bacteria, such as *E. coli*. and *Clostridium perfringens* [24]. Hederacolchiside A1 (HSA), a known structure which contains a trisaccharide scaffold, manifests strong and broad-spectrum antiproliferation inhibitory activities against human cancer cell lines [25]. However, up to now, there is little knowledge about the antischistosomal activity of HSA. In this study, we demonstrate that HSA have antischistosomal activity, affecting parasite viability both in vivo and in vitro.

## 2. Results

### 2.1. HSA Has Antischistosomal Activity against Juvenile and Adult S. japonicum

**Figure 1:**
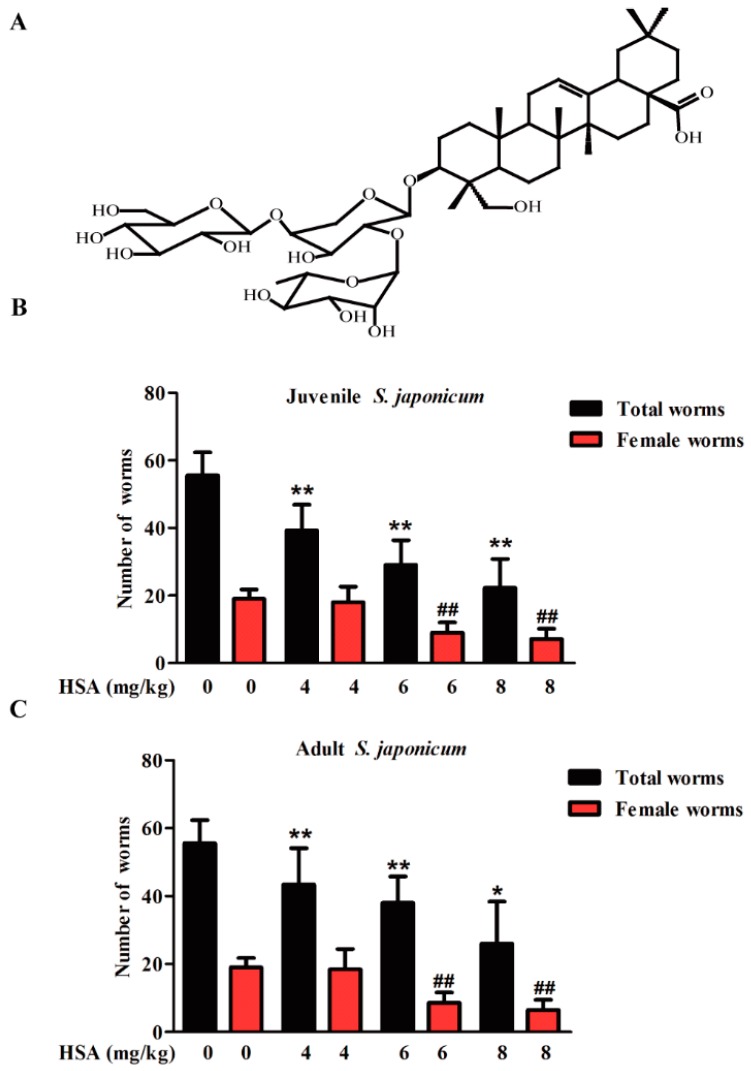
Dose-response relationship of HSA was administered to mice harboring *S. japonicum*. (**A**) Chemical structure of HAS; (**B**) 4–8 mg·kg^−1^ HSA was administered to mice harboring 14-day-old juvenile *S. japonicum*; (**C**) 4–8 mg·kg^−1^ HSA was administered to mice harboring 35 day-old adult *S. japonicum*. Mice were treated by intraperitoneal administration of 4–8 mg·kg^−1^ HSA as shown in Materials and Methods. Infected untreated (Negative control) mice were treated with vehicle. Each bar represents the mean ± SD (* *p* < 0.05, ** *p* < 0.01 vs. total worms of untreated group, *t*-test; ^##^ *p* < 0.01 vs. female worms of untreated group, *t*-test).

Our previous injection toxicity test study found that the mouse median lethal dose (LD_50_) by intravenous injection of HSA is 21.05 mg·kg^−1^. Moreover, mouse could be treated with 8 mg·kg^−1^ HSA by intraperitoneal injection with no obvious toxicity. In view of the promising antischistosomal activity of HSA, total and female worm burden reductions in juvenile (14-day-old) (Figure 1B) and adult (35-day-old) (Figure 1C) *S. japonicum* harbored mice decreased in a time and dose-dependent manner. In the juvenile infection model, total and female worm burden reductions of 59.9% and 62.4% were achieved with 8 mg·kg^−1^ HSA, respectively (Table 1). Moreover, intraperitoneal administration of 8 mg·kg^−1^ HSA on mice infected with adult *S. japonicum* resulted in total and female worm burden reductions of 53.2% and 65.8%, respectively (Table 1). These results revealed the antischistosomal activity of HSA against both juvenile and adult *S. japonicum* with a dose-response relationship.

### 2.2. HSA Was Superior in Inhibiting S. japonicum Less than 11 Days Old

**Figure 2:**
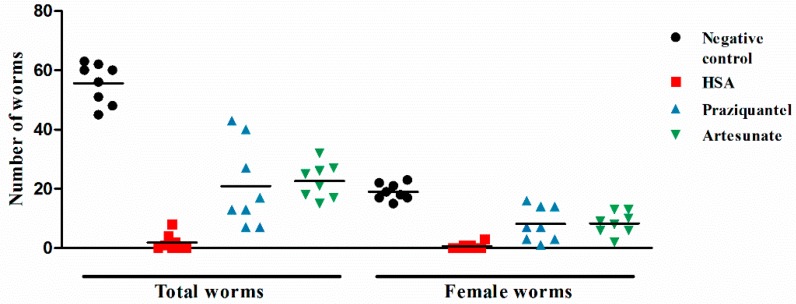
The antischistosomal effect of HSA against 1-day-old juvenile *S. japonicum* harbored in mice. Each point represents data from an individual treated or infected untreated mouse. Horizontal bars indicate mean values. The means ± SD (n = 8) for each experimental condition are as follows: Total (untreated: 55.6 ± 6.8; HSA: 1.9 ± 2.7; praziquantel: 20.9 ± 13.3; artesunate: 22.6 ± 5.5) and female (untreated: 19.0 ± 2.8; HSA: 0.6 ± 1.0; praziquantel: 8.1 ± 5.4; artesunate: 8.4 ± 3.5).

Since 8 mg·kg^−1^ HSA showed the highest activities against juvenile and adult stages of *S. japonicum*, next, a single intraperitoneal dose of 8 mg·kg^−1^ HSA was used to investigate the stage-specific susceptibility of HSA against *S. japonicum*. Table 2 summarizes the antischistosomal activity of HSA shortly after infection (1-day post-infection) and until 49 days post-infection. Regardless of the timing of HSA administration when mice were infected with juvenile or adult *S. japonicum*, total and female worm numbers were reduced highly significant in HSA-treated mice (Table 2). HSA administration to mice harboring 1-, 7-day-old *S. japonicum* showed more effectiveness than other infected stages. More importantly, 8 mg·kg^−1^ HSA was highly active against 1-day-old *S. japonicum* harbored in mice, and the antischistosomal activity of HSA was better than the positive drugs, praziquantel and artesunate (Figure 2). These results demonstrated that HSA showed significant antischistosomal activity on the different development stage of *S. japonicum,* especially for 1-day-old *S. japonicum*.

### 2.3. The Efficacy Advantage of HSA against 1-Day-Old and 7-Day-Old Juvenile S. mansoni

To determine the promising antischistosomal properties of HAS (and due to limited resources), we tested the anti-parasite effect of HSA on juvenile (1-, 7-, 21-day-old) and adult (42-, 49 day-old) *S. mansoni* harbored in mice. As Table 3 shows, HSA resulted in high and comparable total and female worm burden reductions when given to mice infected with either juvenile or adult stages of *S. mansoni*. At a single dose of 8 mg·kg^−1^, HSA achieved worm burden reductions of 88.6 to 80.7% in mice harboring 1-day-old and 7-day-old juvenile *S. mansoni*, respectively. We still observed moderate total and female worm burden reductions of 68.3 to 84.1% in mice harboring 21-day-old juvenile and 49-day-old adult *S. mansoni* treated with a single dose of 8 mg·kg^−1^ HSA (Table 3). Comparing with the untreated group, the significant decrease in total and female worm number was shown in all HSA-treated groups. These results indicated that HSA also had promising antischistosomal properties against 1-day-old and 7-day-old juvenile *S. mansoni*, and that was even better than the positive drugs.

### 2.4. HSA Inhibited Liver Damage in S. japonicum-Infected Mice

Chronic morbidity during infection with *S. japonicum* develops as a result of schistosome eggs that lodge in the liver, gut and other organs, which causes extensive tissue damage [8]. To evaluate the potential effect of HSA on tissue egg loads, liver tissues of infected mice were digested separately in 5% KOH and eggs/g tissues were calculated. The highest reduction percentages of ova in tissues were recorded in HSA-treated mice harboring 1-day-old juvenile *S. japonicum* (99.3%) (Table 4).

**Figure 3:**
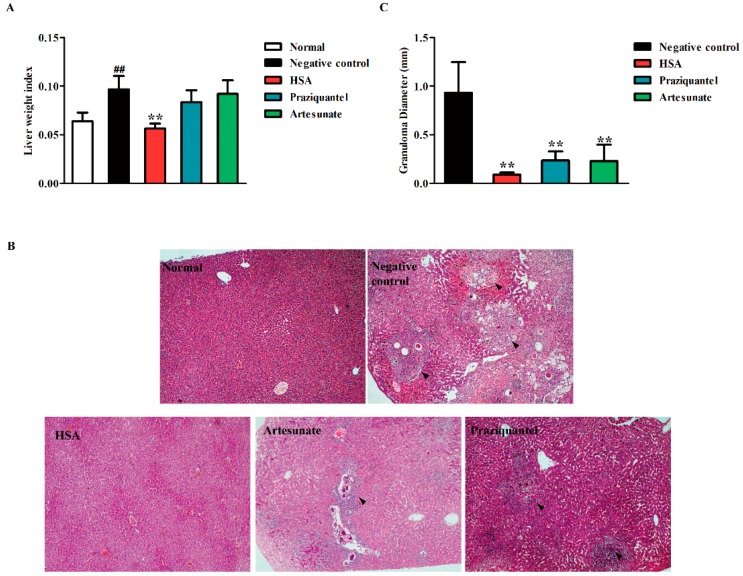
Reduction of hepatic granulomatous inflammation by HSA treated 1–5 days *S. japonicum* post-infected mice. Mice were treated with administration of 8 mg·kg^−1^ HSA, 300 mg·kg^−1^ praziquantel (positive control) and 300 mg·kg^−1^ artesunate (positive control) as antischistosomal treatments for 1-day-old juvenile *S. japonicum* infection. The effect of HSA on hepatic granulomatous inflammation of infected mice was tested. (**A**) Liver weight index (liver weight/body weight). Each bar represents the mean ± SD. ^##^ *p* < 0.01 vs. Normal, *t*-test; ** p < 0.01 vs. Negative control, *t*-test; (**B**) Representative hepatic granulomas of untreated and drug-treated mice. Photographs were taken at 100× (H&E). Black arrows represent the area of granuloma; (**C**) Quantification of egg-induced liver pathology by measurement of mean granuloma diameter. Each bar represents the mean ± SD; ** p < 0.01 vs. Negative control, *t*-test.

To investigate the tissue responses to the HSA treatment, the liver weight index (liver weight/body weight) and liver disease burden (displayed as mean granuloma diameter) of the untreated mice, infected mice, and HSA-treated mice were analyzed. Intraperitoneal administration of HSA at the single doses 8 mg·kg^−1^ with 1-day and 7-day, 14-day and 21-day *S. japonicum* post-infected mice showed a dramatic decrease in the liver index, comparing with the untreated mice (Supplementary Materials Figure S1A). Moreover, HSA achieved lower liver index in mice harboring 1-day-old juvenile *S. japonicum* than praziquantel or artesunate administration (Figure 3A).

Microscopic examination of liver sections stained with hematoxylin and eosin revealed intact liver architecture in all the studied mice groups. The size of granuloma and the intensity of inflammatory infiltrate were evidently variable between the groups in this study (Supplementary Materials Figure S1B). At 7 weeks post-infection, the eggs were surrounded by a dense population of immune cells, such as lymphocytes and eosinophils, followed by a band of fibro-vascular tissue leading to the formation of a mature granuloma in the untreated group (Figure 3B, Black arrows). 

Contrarily, examination of the infected livers revealed a significant reduction in the size of granulomatous inflammation in all HSA-treated mice compared with the untreated mice (Supplementary Materials Figure S1B). Moreover, by administration of 8 mg·kg^−1^ HSA at days 1–5 post-infection, mature granuloma could hardly be detected and the hepatic lobular architecture restored its normal organization and most hepatocytes showed normal appearance (Figure 3B). The reduction degrees of liver disease burden (displayed as mean granuloma diameter) in *S. japonicum*-infected mice after HSA treatment are displayed in Supplementary Materials Figure S1C. The significant changes of granuloma diameter were observed from 0.92 mm in untreated mice to 0.09 mm in 8 mg·kg^−1^ HSA treated 1–5 days post-infected mice (Figure 3C). These results suggested that 8 mg·kg^−1^ HSA could significantly reduce the granulomatous inflammation.

### 2.5. HSA Altered Cytokine Profile in S. japonicum Infected Mice

**Figure 4:**
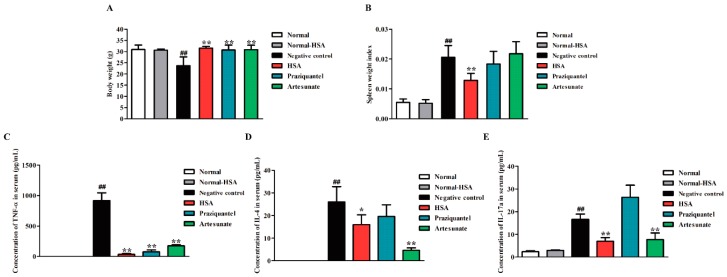
Immune responses in *S. japonicum* infected mice reduced by HSA. Antischistosomal effects on immune responses of 7-week-infected drug-untreated and drug-treated mice. (**A**) Statistical analysis of body weights; (**B**) Spleen weight index (spleen weight/body weight); (**C**) Expression of TNF-α in *S. japonicum* infected mice serum with different treatment; (**D**) Expression of IL-4 in *S. japonicum* infected mice serum with different treatment; (**E**) Expression of IL-17a in *S. japonicum* infected mice serum with different treatment. Each bar represents the mean ± SD. Each bar represents the mean ± SD. ^##^ *p* < 0.01 vs. Normal mice; * p < 0.05 vs. Negative control; ** p < 0.01 vs. Negative control, *t*-test.

In order to investigate the immunomodulatory effect of HSA, we tested the body weight, spleen weight index (spleen weight/body weight) and the expression of Th1/Th2/Th17 cytokines, as the major cytokines responsible for granulomatous inflammatory [26] in HSA treated 1–5 days post-infected mice. For HSA antischistosomal treatment, the body weights of mice in the treatment group were significantly heavier than that of the untreated group, but no significant difference was found between uninfected HSA-treated group and uninfected group (normal control) (Figure 4A). Moreover, the spleen indexes of the mice were also dramatically reduced in the HSA antischistosomal treatment group in comparison to the infected untreated group (Figure 4B).

Schistosome eggs elicit a CD4+ Th cell-mediated hepatic granulomatous inflammation, which is the major pathological consequence of the disease. Granuloma formation is associated with an imbalance in Th1/Th2/Th17 cytokines. In trying to explain the ameliorating effect of HSA on hepatic granuloma size, the serum levels of some cytokines, such as TNF-α, IL-4, and IL-17a, which have been involved in *Schistosoma* granuloma formation in drug-treated mice harboring 1-day-old juvenile *S. japonicum* were measured. Levels of TNF-α (Figure 4C), IL-4 (Figure 4D) and IL-17a (Figure 4E) were significantly decreased in the HSA-treated group, compared with the untreated group. The levels of TNF-α and IL-17a in HSA treated group was lower than praziquantel and artesunate-treated group. These results revealed that immune responses in 1-day-old juvenile *S. japonicum* infected mice were reduced by HSA treatment.

### 2.6. HSA Protects the Liver with Anti-Fibrotic Effects

**Figure 5:**
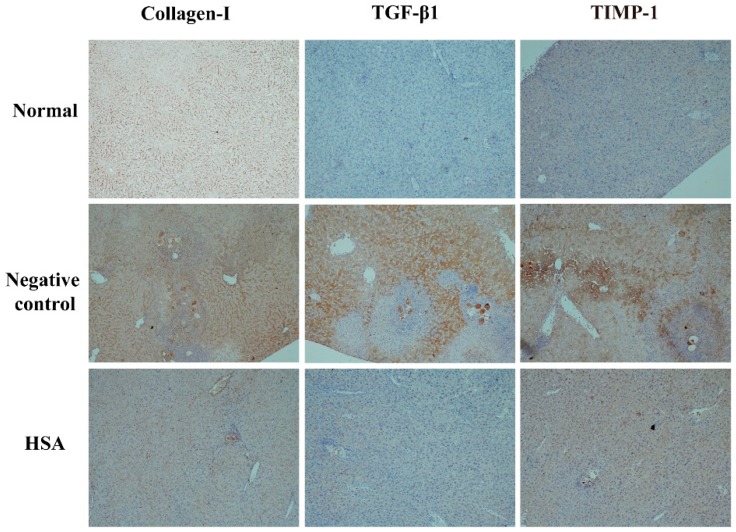
HSA inhibited expressions of fibrotic protein expression in liver of mice. Representative images of immunostaining for TGF-β1, TIMP-1, and Collagen I in 7-week-infected drug-untreated (Negative control) and drug-treated mice (HSA) or uninfected mice (Normal). Target protein positive staining is yellow, brownish-yellow or snuff. Original magnification 100×. The histogram shows integral optical densities of target proteins. TGF-β1: Transforming growth factor-beta 1; TIMP-1: tissue inhibitor of metalloproteinase 1; Collagen I: collagen type I.

In order to examine the anti-fibrotic effect of HSA on 1-day-old *S. japonicum* infected mice, the expression of collagen I, TGF-β1, TIMP-1 in liver tissue was quantified by immunohistochemistry. Wispy traces of collagen I, TGF-β1, TIMP-1 positive staining were sparsely distributed in sections of normal group. At week 7 post-infection, in the untreated group, densely collagen I-, TGF-β1- and TIMP-1-stained cells which could be distinguished by their yellow, brownish-yellow or snuff color surrounded by and infiltrated into the granulomas, and accumulated in fibrotic lesions or stretched along the fibrous septum. In HSA-treated group, the intensity of positive traces was dramatically reduced compared to untreated group; the hepatic lobular architecture was restored to its normal organization and most hepatocytes showed as normal appearance, similar to the one in normal group (Figure 5). These results revealed that HSA inhibited expressions of fibrotic protein expression in liver.

### 2.7. The Inhibition of S. japonicum NTS by HSA Is Partially Due to the Tegumental Disruption

**Figure 6:**
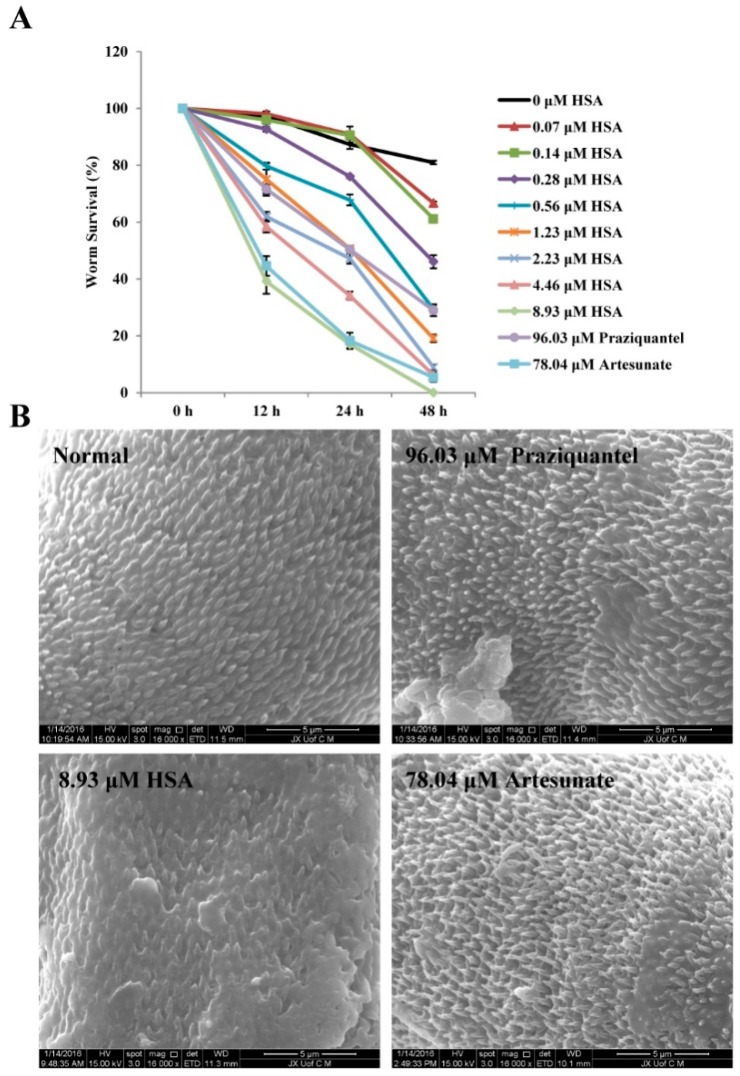
Effects of HSA, praziquantel, and artesunate on NTS *S. japonicum* in vitro. (**A**) Effects of 0–8.93 μM HSA, 96.03 μM praziquantel and 78.04 μM artesunate on NTS *S. japonicum* in vitro. Each point represents the mean ± SD. (**B**) Scanning electron micrograph showing tegument of non-treated NTS *S. japonicum*, 8.93 μM HSA-treated NTS *S. japonicum*, 96.03 μM praziquantel-treated NTS *S. japonicum* and 78.04 μM artesunate-treated NTS *S. japonicum*.

Based on in vivo results, HSA exhibits the highly anti-parasite activity on 1-day-old juvenile *S. japonicum*. Next, we wished to detect the effect of HSA on NTS of *S. japonicum* in vitro. Figure 6A showed that the positive control praziquantel at a concentration of 96.03 μM (30 μg·mL^−1^) killed 70.98% parasites within 48 h; artesunate at a concentration of 78.04 μM (30 μg·mL^−1^) killed 94.59% parasites within 48 h whereas the survival rate of worms belonging to the negative (maintenance medium) control groups was 80.98%. HSA at a concentration of 8.93 μM (8 μg·mL^−1^) killed 100% of parasites after 48 h of incubation. Scanning electron microscopical examination revealed that the dorsal surface of *S. japonicum* NTS worms cultured in negative control medium (maintenance medium) was provided with numerous large tubercles bearing spines after 4 h, while the positive control (96.03 μM praziquantel or 78.04 μM artesunate) had a moderate tegumental alteration in the worms. Interestingly, worm treated with 8.93 μM HSA caused the most serious morphological alterations in the tegument of NTS, especially extensive tegumental disruption such as sloughing and erosion (Figure 6B). These data indicated that the morphological changes in the tegument of the worms induced by HSA might be the antischistosomal mechanism.

## 3. Discussion

Many plants reported to have anthelmintic properties actually contain compounds which are secondary metabolites, such as saponins, alkaloids, non-protein amino acids, tannins and other polyphenols, lignin, glycolides that are directly active against parasites [27,28,29,30]. The gifts from traditional Chinese medicine, artemisinin and its derivatives, were selected for schistosomiasis control [18]. In this study, we discovered the new antischistosomal activity of HSA, a natural product extracted from the *Pulsatilla chinensis* (Bunge) Regel. The data showed that HSA was highly active against less than 11 days old juvenile *S. japonicum* harbored in mice (total and female worm burden reductions ranged between 89.5% and 97.2%. HSA also achieved worm burden reductions from 88.6 to 80.7% in mice harboring less than 11 days old juvenile *S. mansoni*, respectively. It is reported that the relative resistance of the larval stages of *S. mansoni* to schistosomicidal drugs might be a result in a therapeutic failure because of the presence of migrating, drug-resistant, immature forms of the parasite [31]. In this regard, HSA may be a potential drug of choice for the prevention and treatment of *S. japonicum* and *S. mansoni* infections. 

The reduction in the female worm recovery and egg load in treated mice was considered as a strong evidence of the efficiency of antischistosomal drugs. Indeed, the significant improvement parameters after treatment of mice at days 1–5 post-infection of *S. japonicum* with 8 mg·kg^−1^ HSA resulted in the significant reduction in female worm burdens (94.7%) accompanied with a significant decrease in the percentage of ova load (99.3%) compared with the control group. Moreover, the increase in the relative liver weight may be attributed to both egg deposition by worms and several metabolites released by *S. mansoni*, which affect the host hepatic tissue [32]. In this study, intraperitoneal administration of HSA at the single doses 8 mg·kg^−1^ to 1-day, 7-day, 14-day and 21-day *S. japonicum* post-infected mice showed a dramatic decrease in the liver index, comparing with the untreated mice. Moreover, HSA achieved lower liver index in mice harboring 1-day-old juvenile *S. japonicum* than praziquantel or artesunate administration.

Chronic morbidity during infection with *S. japonicum* and *S. mansoni* develops as a result of schistosome eggs that lodge in the liver, gut and other organs, which causes extensive tissue damage, such as granulomatous inflammation and tissue fibrosis [8]. Therefore, reducing egg counts in the tissues can significantly relieve the symptoms of schistosomiasis [33,34]. On histopathological examination of the liver of HSA-treated mice, there were either active small granulomas or healed granulomas. By administration of 8 mg·kg^−1^ HSA on 1–5 days post infected mice, mature granuloma could hardly be detected and the hepatic lobular architecture restored its normal organization and most hepatocytes showed normal appearance. The reduction degree of liver disease burden (displayed as mean granuloma diameter) in *S. japonicum*-infected mice after HSA treatment was consistent with the above results. 8 mg·kg^−1^ HSA treated 1–5 days post-infected mice could dramatically reduce the granuloma diameter, from 0.92 mm in control group to 0.09 mm in the HSA-treated group. These results suggested that HSA has a considerable effect on *Schistosoma* pathological changes in the liver. This could be attributed partly to the reduction in the number of eggs trapped in the hepatic tissues and the modulation of serum levels of some cytokines, which are incriminated in the development of *Schistosoma* granuloma.

Indeed, granuloma formation is dependent on CD4^+^ Th cell response. *Schistosoma* antigens manifest a striking shift from a moderate Th1 to a robust Th2-dominated response with the onset of egg lying around 5–6 weeks [26,35,36]. In order to confirm the ameliorating effect of HSA on hepatic granuloma size, the serum levels of Th1/Th2/Th17 cytokines, which have been involved in Schistosoma granuloma formation, were measured. We found that the levels of TNF-α, IL-4, and IL-17a were significantly decreased in HSA 1-day-treated group, compared with the untreated group. Importantly, the levels of TNF-α and IL-17a in HSA treated groups were lower than praziquantel and artesunate-treated group. These results demonstrated HSA treatment promoted a significant and high decrease in granuloma formation as well as in the immune response that underlies granuloma development.

Previous investigate showed that prolonged Th2 [37] and Th17 [38] responses contributed to the development of hepatic granulomatous inflammation and hepatic fibrosis. The mechanism of resulting liver fibrosis is the same that is the activation of Hepatic stellate cells (HSCs) and subsequent extracellular matrix deposition. HSCs, one of the main sources of collagen in the liver, play a crucial role in schistosome-induced fibrogenesis [39]. Chemokines associated with HSCs recruitment and activity, these activated HSCs localized to the granulomas, thus serve as an indicator of collagen deposition in hepatic schistosomiasis [40]. Deposition of collagen, imbalance of matrix metalloproteinase/tissue inhibitor of metalloproteinase (TIMP) and secretion of profibrotic cytokines caused by activated HSCs were described previously in schistosomiasis [41,42]. In additional, TGF-β1 promotes collagen synthesis in activated HSCs via pSmad2/3 pathways [43]. To investigate the anti-fibrosis activity of HSA, we detected the expressions of the fibrosis-related marker, collagen Ⅰ, TGF-β1 and TIMP-1 in infected mice. There was no fibrosis-related marker in the HSA-treated group. Therefore, these results demonstrated that hepatic fibrosis was hardly detected in *S. japonicum*-infected mice after HSA anti-parasite therapy. In our experiments, the anti-fibrosis effect of HSA might be partly due to its anti-parasite activity, and the anti-inflammation property of HSA would study further.

Based on in vivo results, HSA exhibits the significant anti-parasite effect on 1-day-old *S. japonicum,* we wonder to detect the effect of HSA on NTS of *S. japonicum* in vitro. In the present study, HSA at a concentration of 8.93 μM killed 100% of NTS *S. japonicum* worms after 48 h of incubation; however, the 100% lethal concentration for positive control, praziquantel, and artesunate, in the same culture time was higher. The tegument of schistosomes is an important target for antischistosomal drugs [44]. Various antischistosomal drugs such as praziquantel [45], artemether and artesunate [46,47], mefloquine [48], miltefosine [49], epiisopiloturine [50] and piplartine [51] have been documented for alterations in the tegument of schistosoma species. In the absence of the drug, NTS of *S. japonicum* showed normal viability without any tegumental changes for 4 h. In the presence of HSA at concentrations of 8.93 μM, extensive tegumental disruption such as sloughing and erosion was observed by SEM examination, while the positive control (96.03 μM praziquantel or 78.04 μM artesunate) had a moderate tegumental alteration in the worms. These in vitro results were consistent with the antischistosomal activity of HSA on 1-day-old juveniles chistosomes harbored in mice.

In all, our data demonstrated for the first time that HSA, the natural product isolated from the *Pulsatilla chinensis* (Bunge) Regel, exhibits antischistosomal properties in vivo against *S. japonicum and S. mansoni*. The antischistosomal activity is higher than positive drugs, praziquantel, and artesunate against 1-day-old juvenileschistosomes. In addition, 8 mg·kg^−1^ HSA ameliorate the pathological parameters, such as granuloma formation, granulomatous inflammation and liver fibrosis in the *S. japonicum* infected mice. Furthermore, the antischistosomal activity of HSA on NTS confirmed the in vivo results. Further in vitro and in vivo studies would be launched to elucidate the possible mechanism of action and to study the effect of HSA on schistosomes and other trematodes. These results suggest that HSA may have the possibility to be a useful antischistosomal agent for therapy in human schistosomiasis and provide a basis for future clinical trials.

## 4. Materials and Methods

### 4.1. Animals

ICR mice of similar age and weight (20–25 g) were used for this study. They were purchased from the Experimental Animal Center of Soochow University (Suzhou, China) and were housed under specific pathogen-free conditions. The animal room was controlled under temperature (22 ± 2 °C), light (12 h light/dark cycle) and humidity (50 ± 10%). All laboratory feed pellets and bedding was autoclaved. The mice were anesthetized using diethyl ether and blood samples were withdrawn from the tail vein of mice. The animal study proposal was approved by the Institutional Animal Care and Use Committee of the Soochow University. Experimental procedures involving animals were performed in accordance with the Regulations for the Administration of Affairs Concerning Experimental Animals approved by the State Council of People’s Republic of China.

### 4.2. Compounds

HSA (2.5 g) was prepared in our lab, and the structure (Figure 1) was identified by comparison its spectroscopic data with those of HSA, which are in agreement with those of *Pulsatilla* saponin B7 [52]. The purity of HSA was determined as 95.2% by analytical HPLC with PDA detection.

### 4.3. In Vivo Studies with S. japonicum

Mouse (eight per group) was infected percutaneously with ~65 *S. japonicum* cercariae. To investigate the dose-response relationship of HSA against both the juvenile and adult *S. japonicum*, 4–8 mg/kg intraperitoneal doses were given to mice 14 days (pre-patent infection) and 35 days (patent infection) post-infection for five consecutive days. To assess the efficacy of HSA against different stages of *S. japonicum*, mice were intraperitoneally treated with a single dose of 8 mg·kg^−1^ HSA daily for 5 consecutive days. Different treatment groups started at either of day 1, 7, 14, 21, 28 and 35 post-infection. In each experiment, infected but untreated mice served as controls. For comparison, praziquantel (300 mg·kg^−1^) (Sigma-Aldrich Chemie GmbH, St. Louis, MO, USA) or artesunate (300 mg·kg^−1^) (batch no. 20081213; 99.9% purity) were orally administered with infected mice as the positive control group. At 49 days post-infection, mice were killed and the worms were recovered from the hepatic and port mesenteric veins by the perfusion technique [53].

### 4.4. In Vivo Studies with S. mansoni

Mouse (eight per group) was infected subcutaneously with ~80 S. mansoni cercariae. To study the stage-specific susceptibility of S. mansoni, mice were treated intraperitoneal with a single 8 mg·kg^−1^ dose of HSA for 5 consecutive days. Each group started treatment at either of day 1, 7, 21, 42 and 49 post-infection. For each experiment, infected but untreated mice served as negative control. Praziquantel (300 mg·kg^−1^) or artesunate (300 mg·kg^−1^) were orally administered with infected mice as the positive control. At 56 days post-infection, worms were recovered from the hepatic perfusion as described elsewhere [54,55].

The percentage of reduction in worm numbers after treatment was calculated according to Tendler et al. [56] as follows: P = (C − V/C) × 100, where P = percentage of worm burden, C = mean number of parasites recovered from infected but not treated animals, and V = mean number of parasites recovered from the treated animals.

The animals were then sacrificed and their livers were separated. Egg count in the liver was demonstrated by taking a weighted portion of the liver and each placed in a test tube containing 5 mL of 5% KOH solution [57]. Eggs were counted after being spread on slides and the number of eggs per tissues weight (gram) was calculated. The calculation of egg reduction rate was similar to the worm burden rate.

### 4.5. Histology Analysis

All mice were weighed and sacrificed. Liver samples were weighted, fixed in 4% formalin, paraffin embedded and sectioned (4 mm thick). All sections were stained with hematoxylin and eosin to evaluate structural alterations of the hepatic parenchymal cells and to clarify the presence of schistosome eggs and granuloma.

Liver disease burden (displayed as mean granuloma diameter) was measured for individual mice, and the results were reported as the mean with standard deviation of the group. To measure these, six random photographs (CX31, × 40 magnification, Olympus, Tokyo, Japan) were taken from each of three randomly cut liver sections per mouse, the mean diameter (mm) was measured by image analysis software (Image J, NIH, Bethesda, MD, USA). Counts for each photograph were averaged over photographs within animals. The means and standard deviations were calculated for each group.

### 4.6. Immunological Analysis

Mice were anesthetized using diethyl ether after measured the weight of each mouse and then, blood was collected from the sublingual vein. The animals were then sacrificed and their spleens were separated. Blood was obtained from each mouse after sacrifice. Serum was collected from the clotted blood samples after centrifugation at 400× *g* for 15 min at 4 °C, then divided into aliquots and stored at −80 °C until use.

Cytokines interleukin (IL)-4, tumor necrosis factor (TNF)-α and IL-17a were measured in the sera of mice by using sandwich ELISAs with anti-cytokine antibodies according to the manufacturer’s instructions. BMS613 Mouse IL-4 platinum ELISA, BMS607/3 Mouse TNF-α platinum ELISA, BMS6001 Mouse IL-17a platinum ELISA were purchased from eBiosience (San Diego, CA, USA).

### 4.7. Immunohistochemistry Analysis

Immunohistochemical staining was performed with an HRP-Polymer anti-Mouse/Rabbit IHC Kit (GTX83398, Irvine, CA, USA) The sections were deparaffinized, washed in phosphate-buffered saline (PBS, 0.01 mol·L^−1^, pH 7.2) 3 × 5 min, heated at 100 °C in a microwave oven 6 × 2 min, incubated in 3% H_2_O_2_ in deionized water for 10 min to block endogenous peroxides activity, and washed 3 × 5 min with PBS. The sections were then incubated overnight at 4 °C with the following primary antibodies: anti-transforming growth factor-β 1 (TGF-β1) antibody (ab92486, Abcam, Cambridge, MA, USA, 1:500); anti-tissue inhibitor of metalloproteinase-1 (TIMP-1) antibody (ab61224, Abcam, 1:200); anti-collagen I antibody (ab34710, Abcam, 1:500). After washing 3 × 5 min with PBS, the appropriate HRP-polymer anti-mouse/rabbit immunoglobulin G was added to the sections and incubated at 37 °C for 20 min. The sections were then washed 3 × 5 min with PBS, and the color was developed with DAB for 3–5 min. The nuclei were lightly counterstained with hematoxylin. 

### 4.8. Cultivation of Newly Transformed Schistosomula (NTS-the Larval Stage) S. japonicum and Scanning Electron Microscopy (SEM)

#### 4.8.1. Collection of NTS *S. japonicum*

NTS were obtained using a transformation method described previously [58]. Briefly, the collected cercarial suspension was cooled, centrifuged and pipetted, and vortexed vigorously in Hanks’s balanced salt solution (HBSS) to remove the tails. The NTS suspension was adjusted to a concentration of 100 NTS per 50 μL in NTS culture medium, the RPMI 1640 medium (Invitrogen, Carlsbad, CA, USA) (maintenance medium) containing 10% fetal bovine serum, 100 U·mL^−1^ penicillin, 100 μg·mL^−1^ streptomycin (Invitrogen). The NTS suspension was then incubated at 37 °C, 5% CO_2_ in ambient air for 12 h.

#### 4.8.2. Schistosome Incubation In Vitro with Treatment

Twelve hours after NTS preparation, approximately 150 *S. japonicum* NTS per well were cultured in 6 well plate with 4 mL maintenance medium. The *S. japonicum* were then treated with different concentrations of HSA (0–8.93 μM), 96.03 μM praziquantel or 78.04 μM artesunate for 72 h.

#### 4.8.3. SEM

The *S. japonicum* NTS were selected for SEM according to the morphology of worms. Approximately 10 *S. japonicum* NTS were cultivated in maintenance medium containing 8.93 μM HSA, 96.03 μM praziquantel or 78.04 μM artesunate for 4 h and then sequentially fixed in 10% formaldehyde buffer at 4 °C for 4 h, osmium tetroxide phosphate buffer solution (1%) at 4 °C for 2 h. After washing with phosphate buffer solution, the schistosomes were dehydrated in ascending grades of alcohol and critical-point dried in liquid carbon dioxide. Finally, the samples were sputter-coated with gold and examined by SEM (Quanta 250, FEI, Hillsboro, OR, USA).

### 4.9. Statistical Analysis

Means of multiple groups were compared using one-way ANOVA followed by Tukey’s multiple comparisons test employing Prims software (GraphPad Software, La Jolla, CA, USA). Data were expressed as mean ± standard deviation (SD). The data were considered significant if *p* < 0.05.

## Figures and Tables

**Table 1:** Total and female worm burdens in the different dosage of HSA, praziquantel or artesunate treatment against the schistosomula and adult worms of *S. japonicum*.

Treatment (Dose mg·kg^−1^)	Worm Reduction Rate (%)			
	Total	Female	Total	Female
	14–18 days		35–39 days	
Control	__	__	__	__
HSA (8)	59.9	62.4	53.2	65.8
HSA (6)	47.6	52.6	32.4	52.6
HSA (4)	29.3	5.3	21.8	2.9
praziquantel (300)	52.0	45.6	85.2	83.2
artesunate (300)	85.1	85.0	92.4	94.7

**Table 2:** Stage-specificity of a single 8 mg·kg^−1^ dose HSA, praziquantel or artesunate was administered to mice infected with *S. japonicum*.

Treatment Stage of Post-Infection	Treatment (Dose mg/kg)	Mean Number of Worms (SD)		Worm Burden Reduction (%)	
		Total	Female	Total	Female
1–5 days	-	55.6 (6.8)	19.0 (2.8)	—	—
	HSA (8)	1.9 (2.7) ***	0.6 (1.0) ***	97.2	94.7
	praziquantel (300)	20.9 (13.3) ***	8.1 (5.4) ***	62.4	57.2
	artesunate (300)	22.6 (5.5) ***	8.4 (3.5) ***	59.3	55.9
7–11 days	HSA (8)	5.5(3.3) ***	1.6(3.3) ***	90.0	89.5
	praziquantel (300)	22.4(6.8) ***	7.6(2.9) ***	55.6	59.9
	artesunate (300)	26.4(11.9) ***	10.0(5.0) **	52.5	47.4
14–19 days	HSA (8)	22.3 (8.6) ***	7.2 (3.1) ***	60.7	63.2
	praziquantel (300)	26.7 (9.5) ***	10.3 (3.2) ***	52.0	45.6
	artesunate (300)	8.3 (5.9) ***	2.9 (2.2) ***	85.1	85.0
21–25 days	HSA (8)	24.6 (9.6) ***	6.4 (4.1) ***	55.4	68.4
	praziquantel (300)	7.9 (5.8) ***	5.0 (3.5) ***	85.9	73.7
	artesunate (300)	8.7 (3.8) ***	5.4 (2.8) ***	84.3	71.4
28–32 days	HSA (8)	32.6 (7.1) ***	6.1 (1.8) ***	41.1	68.4
	praziquantel (300)	4.3 (3.4) ***	3.5 (2.5) ***	92.2	81.6
	artesunate (300)	1.5 (1.9) ***	1.3 (1.6) ***	97.3	93.0
35–39 days	HSA (8)	26.0 (12.4) **	6.5 (3.1) ***	53.2	65.8
	praziquantel (300)	8.2 (14.9) **	3.2 (5.9) **	85.2	83.2
	artesunate (300)	4.3 (4.0) ***	1 (1.2) ***	92.4	94.7

** *p* < 0.01 vs. the negative control group, *t*-test; *** *p* < 0.001 vs. the negative control group, *t*-test.

**Table 3:** Stage-specificity of a single 8 mg·kg^−1^ dose HSA, praziquantel or artesunate was administered to mice infected with *S. mansoni*.

Treatment Stage of Post-Infection	Treatment (Dose mg/kg)	Mean Number of Worms (SD)		Worm Burden Reduction (%)	
		Total	Females	Total	Females
1–5 days	-	38.3 (16.6)	14.1 (7.8)	—	—
	HSA (8)	4.4 (2.7) ***	1.0 (1.5) **	88.6	92.9
	praziquantel (300)	5.3 (5.4) ***	2.3 (2.6) **	86.2	83.8
	artesunate (300)	13.6 (4.7) **	5.0 (1.8) *	64.4	64.6
7–11 days	HSA (8)	7.4 (3.1) **	2.1 (1.5) **	80.7	85.0
	praziquantel (300)	13.2 (6.3) **	2.5 (1.5) **	65.6	82.3
	artesunate (300)	23.8 (11.2)	6.5 (3.6) *	37.7	54.0
21–25 days	HSA (8)	12.1 (7.0) **	4.3 (3.3) *	68.3	70.0
	praziquantel (300)	16.7 (10.3) *	5.0 (3.7) *	56.4	64.6
	artesunate (300)	1.5 (1.3) ***	0.2 (0.4) **	96.1	98.8
42–46 days	HSA (8)	9.5 (4.4) **	2.3 (1.4) **	75.2	84.1
	praziquantel (300)	13.2 (8.5) *	4.5 (3.5) **	65.6	68.1
	artesunate (300)	2.5 (2.5) ***	0.7 (0.7) **	93.5	95.3
49–53 days	HSA (8)	11.3 (4.1) **	2.5 (1.4) **	70.6	82.3
	praziquantel (300)	1.3 (0.7) **	0.3 (0.5) ***	96.5	97.6
	artesunate (300)	13.7(7.0) **	3.8 (2.0) **	64.3	72.9

* *p* < 0.05, ** *p* < 0.01 *** *p* < 0.001 vs. the Negative control group, *t*-test.

**Table 4:** Ova load in *S. japonicum* infected mice received HSA, praziquantel or artesunate treatment.

Treatment Stage of Post-Infection	Treatment (Dose mg/kg)	Hepatic Ova Reduction Rate (%)
1–5 days	-	—
	HSA (8)	99.3
	praziquantel (300)	64.7
	artesunate (300)	61.3
7–11 days	HSA (8)	98.7
	praziquantel (300)	68.1
	artesunate (300)	54.6
14–19 days	HSA (8)	72.6
	praziquantel (300)	59.5
	artesunate (300)	97.6
21–25 days	HSA (8)	79.7
	praziquantel (300)	89.9
	artesunate (300)	89.6
28–32 days	HSA (8)	75.7
	praziquantel (300)	98.2
	artesunate (300)	99.8
35–39 days	HSA (8)	75.4
	praziquantel (300)	97.8
	artesunate (300)	99.7

## Supplementary Materials

The following are available online.

## Abbreviations

HAS, Hederacolchiside A1; HSCs, Hepatic stellate cells; NTS, newly transformed schistosomula; Th cell, T-helper cell; TIMP-1, tissue inhibitor of metalloproteinase-1.
