# Supplementary material for: Antischistosomal Properties of Hederacolchiside A1 Isolated from Pulsatilla chinensis
Source: Molecules. 2018 Jun 13;23(6):1431. doi: 10.3390/molecules23061431 (PMC6099544; doi:10.3390/molecules23061431)
Supplement: Supplementary file 1 [file molecules-23-01431-s001.pdf]

Supplementary Fig. S1

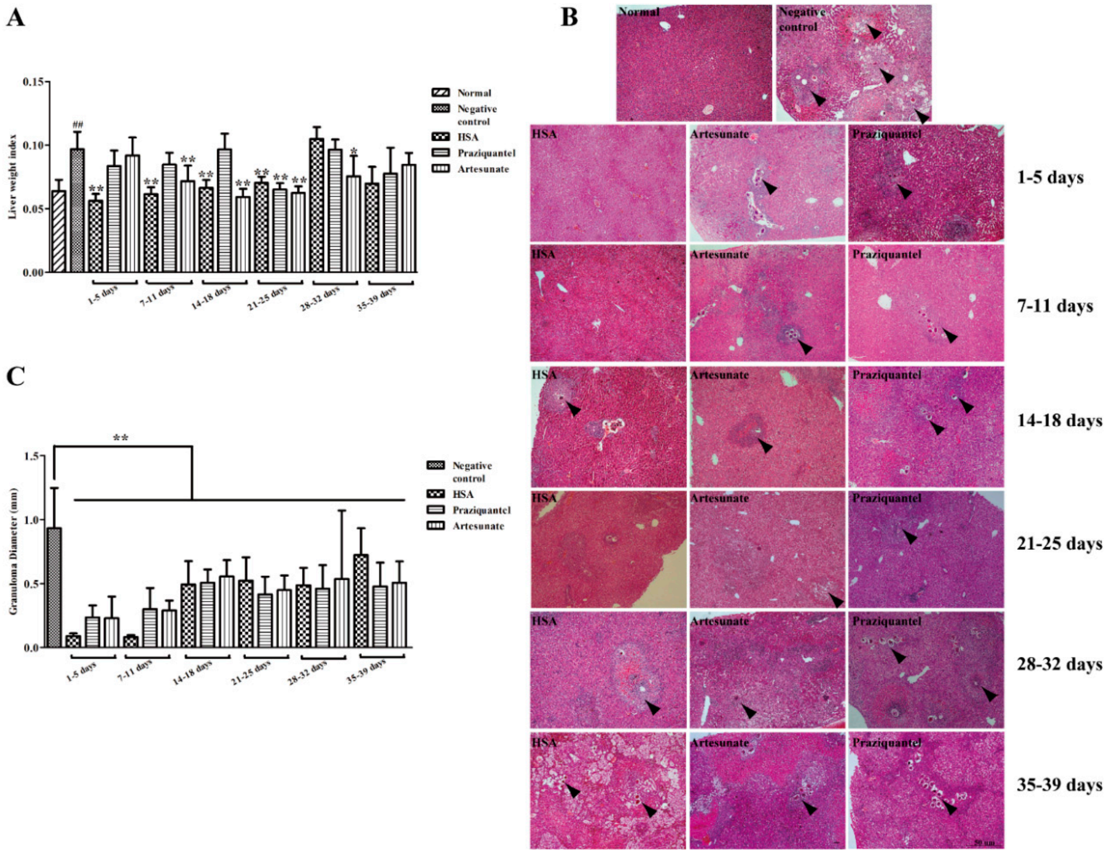

Figure S1. Reduction of hepatic granulomatous inflammation by HSA in mice. Mice were treated by intraperitoneal administration of 8 mg·kg^{-1} HSA, 300 mg·kg^{-1} praziquantel (positive control) and 300 mg·kg^{-1} artesunate (positive control) as antischistosomal treatments for *S. japonicum* infection as shown in Materials and Methods. The effect of HSA on hepatic granulomatous inflammation of infected mice was tested. A. Liver weight index (liver weight/body weight). Each bar represents the mean ± SD. $^*P<0.05$ vs Negative control, T. test; $^{**}P<0.01$ vs Negative control, T. test). B. Representative hepatic granulomas of 7-week-infected drug-untreated and drug-treated mice. Photographs were taken at 100× (H & E). Black arrows represent the area of granuloma. C. Quantification of egg-induced liver pathology by measurement of mean granuloma diameter. Each bar represents the mean ± SD; $^{**}P<0.01$ vs Negative control, T. test.
